# Microbiota shows major difference in case of two shorebird species with different feeding strategy

**DOI:** 10.1016/j.vas.2026.100754

**Published:** 2026-06-26

**Authors:** Ákos Őrsi, Levente Laczkó, Renáta Bőkényné Tóth, Csongor Freytag, Pál Tóth, Gábor Simay, Nándor Szabó, Gábor Kardos, Ádám Lovas-Kiss

**Affiliations:** a MTA-HUN-REN-Centre for Ecological Research “Momentum” Lendület Dispersal Ecology Research Group, Debrecen, Hungary; b Department of Planetary Health, “One Health” Institute, Faculty of Health Sciences, University of Debrecen, Egyetem tér 1, 4032 Debrecen, Hungary; c Department of Infection Control and Hospital Epidemiology, “One Health” Institute, Faculty of Health Sciences, University of Debrecen, Egyetem tér 1, 4032, Debrecen, Hungary; d Department of Bioinformatics, “One Health” Institute, Faculty of Health Sciences, University of Debrecen, Egyetem tér 1, 4032. Debrecen, Hungary; e HUN-REN-DE Conservation Biology Research Group, University of Debrecen, Egyetem tér 1, 4032, Debrecen, Hungary; f Center for Metagenomics, University of Debrecen, Egyetem tér 1, 4032, Debrecen, Hungary; g Hortobágyi National Park Directorate, Debrecen, Hungary; h Pál Juhász-Nagy Doctoral School, University of Debrecen, Egyetem tér 1, 4032, Debrecen, Hungary

**Keywords:** Microbiome, Shorebird, Fusobacteria, Bird migration, 16S rRNA gene, Waterbird, Pathogens

## Abstract

Despite the well-known effects of the gut microbiota on mammals, other vertebrates have only recently begun receiving attention in research. Our study focused on describing the cloacal microbiome of Common Snipe (*Gallinago gallinago*) and Wood Sandpiper (*Tringa glareola*), using 16S rRNA metabarcoding, to understand how different foraging methods can affect their microbiome. Assessing the host microbial diversity, we found that Shannon- (W = 253, p = 0.099), Simpson- (W = 268, p = 0.168) and inverse Simpson- diversities (W = 268, p = 0.168) did not differ significantly, however, there was a tendency towards the Wood Sandpiper having the higher values. SIMPER analysis revealed that the differences were caused by several bacterial taxa, the biggest contributor being *Catellicoccus marimammalinum* (mean contribution = 2.76%, p = 0.003) which had greater abundances in Common Snipe (mean relative abundance = 22.76%) than in the Wood Sandpiper (8.27%). We found great differences in *Fusobacteria* abundances between the hosts, as this phylum had an average abundance of 29.4% in Wood Sandpiper and 8.8% in Common Snipe samples. This difference in their microbiome may be explained by the higher chitin consumption of Wood Sandpiper which is associated with higher *Fusobacteria* abundance. We found multiple important animal (*Mycoplasma iowae, Brachyspira hyodysenteriae*) and human pathogens (*Campylobacter jejuni, Aeromonas veronii, Vibrio cholerae*), some of which are also associated with the growing problem of antimicrobial resistance (*Escherichia coli, Enterococcus faecalis*). The high prevalence of these pathogens in wild waterbirds should be considered important when assessing human and environmental health hazards.

## Highlights

• Two shorebirds with similar foraging habits show slight differences in microbiome composition.
• Differences observed may be a result of differing feeding techniques and food choices.
• Wood sandpiper had substantially higher Fusobacteria abundance, contrary to expectations.
• Shorebirds may be important carriers of both animal- and human pathogens, thus needed to be considered when assessing health hazards.

## 1 Introduction

Migratory birds can be carriers of a wide variety of microorganisms both internally and externally, thus having the potential to transfer them over great distances. They can be both biological- and mechanical carriers and birds are speculated to host many infections not pathogenic to them, like foot- and mouth disease virus, or different *Campylobacter* species (Hubálek, 2004). Birds may carry microorganisms in the gut microbiome for prolonged periods (Bodawatta et al., 2022), including potentially drug-resistant bacteria. Multiple studies revealed that birds can be the carriers of different kinds of antibiotic resistant bacteria, thus migratory birds pose a potential health hazard, as they can spread these microbes over large geographical distances in a short period of time, contributing to their long-distance dispersal (Bonnedahl & Järhult, 2014; Yuan et al., 2021). Studies also revealed that bird microbiomes are largely dependent on external factors such as food and environment, but they also harbour a smaller number of long-term colonizers in their gastro intestinal (GI) tract (Bodawatta et al., 2021). The gut microbiome is known to have various functions in birds which are still only partly understood, but are known to help with nutrient uptake, detoxification, and immune function (Grond et al., 2018). Revealing the interactions between the gut microbiota and the host is a key in understanding the hazards posed to humans, and the role the microbiome plays in the life of the host.

In recent years some studies have described indications of possible phylosymbiotic links between the host and the gut bacteria of birds (Capunitan et al., 2020; Kropáčková et al., 2017). Kropáčková et al. (2017) described significant correlation between host’s phylogenetic divergence and the divergence of the gut microbiome composition, although, according to Capunitan et al. (2020) a White Noise model fits this relation the best, indicating a weak link between microbiome and host’s phylogenetic divergence. In some instances no phylosymbiosis was found (Bodawatta et al., 2021), however the differences in microbiome among birds cannot be explained solely by the ecological divergence of the host populations (Kropáčková et al., 2017). To assess these contradictory results, further research will be required, considering that extrapolating results obtained on *Passerines* is not necessarily representative when researching other groups, because body size has been shown to have a significant effect on the microbiome. Hosts with larger body mass have a significantly smaller intraspecific variation and amplicon sequencing variant (ASV) richness. This could be a result of a more stable environment inside the gut, caused by a longer retention time (Bodawatta et al., 2021). The evolutionary adaptation of flight in birds and bats led to shortened intestinal tracts and reduced retention times, likely minimizing flight load (Caviedes-Vidal et al., 2007). The highly stable bacterial communities across diverse mammalian taxa may be the exception and not the rule, as no other group has been shown to present a similar level of stability and strong correlation with the host’s phylogeny in their microbiome (Song et al., 2020).

The gut microbiome of vertebrates is known to have an important role in host development and physiology. In mammals, the effect of microorganisms in the gastrointestinal tract is known to be ranging from influencing the immune system and nutrient uptake, to playing a major role in GI tract morphology and general cellular functions (Leser & Mølbak, 2009). Despite the known effects on mammalian host health, studies on bird microbiota lags behind. According to Grond et al. (2018), research on gut microbiota is dominated by mammalian studies, outnumbering those of birds in a ratio of 10:1. Research of wild birds are an even smaller minority, as most research focuses on domestic poultry. Considering that *Galloanserae* diverged from *Neoaves* around 90 million years ago (Jarvis et al., 2014) these studies are unlikely to be a good representation for most of the wild bird populations (Bodawatta et al., 2022).

In order to understand the basic functions of wild birds’ gut microbiota, and the health risks they may pose to humans, it is essential to investigate the environmental, ecological and phylogenetical traits of the host and taking these variables into account when looking at the microbial composition. Seasons (Schmiedová et al., 2023) and foraging ecology (Bodawatta et al., 2021) can have substantial effect on the microbiome composition and diversity. Schmiedová et al. (2023) described a marked effect of rainy and dry seasons on tropical *Passerines’* gut microbiota, the difference being larger then between the sampled temperate and tropical birds, separated by thousands of kilometres. Looking at birds with similar, but not completely identical foraging niches may be a good way to further explore the cause of microbial differences in wild birds, as environmental conditions may have a decisive effect on the gut microbiome (Schmiedová et al., 2023). The Common snipe (*Gallinago Gallinago*) and the Wood sandpiper (*Tringa glareola*) are two suitable species for this task, as they have similar foraging habits (Cramp & Simmons, 1983) and use the same freshwater stopover sites during their migration through Hungary, thus it is possible to sample them in the same area, reducing the effect of spatial variation.

Our first aim was to describe the gut microbial community of these two birds. The available data on wild birds’ microbiota are very limited, and it is important to establish a reference database. This could help with the understanding of basic microbial functions in the avian gut, or later creating health monitoring techniques based on microbial markers (Bodawatta et al., 2022). The second objective was to make a comparison of the two species’ microbiome, in order to help getting a basic insight into what role does the host foraging niche has on the gut microbial community. We hypothesised that smaller differences in their food choices and feeding techniques could cause a noticeable difference in their microbiome, and investigating these smaller variations could help with understanding different microbial functions. Both species have a similar, probing technique when looking for prey, however, *G. gallinago* is known to use its long bill to search for prey in slightly deeper waters, relying less on visual prey discovery. Both are common breeding birds of Northern Europe (Cramp & Simmons, 1983) (Chisholm, 2007), but are relatively common species during their migration period in the Central European region, making them suitable subjects for this comparison. The wintering sites and migratory strategies are also different for the two species (Wlodarczyk et al., 2007) making this the other interesting difference worth further investigation, when looking at the microbiome.

## 2 Methods

### 2.1 Study species

*T. glareola* is a common breeding species in Northern-Europe, despite the population decline outside of this area in recent decades, due to habitat destruction in the wetland areas all across Europe (Chisholm, 2007). Being a long-distance migrant species, it moves during autumn migration to its trans-Saharan wintering sites in West-, Central- (Meltofte, 1996) and South-Africa (Wlodarczyk et al., 2007). *T. glareola* uses Hungarian wetlands as stopover sites both during their autumn and spring migration. According to the categorization system of Alerstam and Högstedt (Alerstam & Högstedt, 1982, Wlodarczyk et al., 2007) *T. glareola* is an S-strategist migrant, meaning it has few stopover sites and covers long distances at once.

When staying in the inland water bodies that serve as the stopover sites, the sandpipers were observed to spend around 90% of daytime foraging, to accumulate fat reserves for the long stretches of flight (Krupa et al., 2009). As it is typical for the *Scolopacidae* family, *T. glareola* has medium-long bill and relatively small eyes. These morphological traits are usually associated with tactile, or not highly specialized feeding (Durell, 2000), although these habits may vary depending on age, sex, and the habitat conditions (Krupa et al., 2009). Previously Krupa et al. (Krupa et al., 2009) described *T. glareola* not only probing in deeper water, but frequently pecking on the surface and moving swiftly after prey. This foraging pattern suggests a high dependence on visual information during the search for food, which shows a lower level of specialization.

*G. gallinago* is mainly found in temperate and lower arctic zones in the Western- Palearctic region. These breeding populations in Europe prefer shallow water, dense vegetation with open patches of sedges, rushes and course grasses. This mixture indicates ideal soil conditions for the probing foraging habit (Cramp & Simmons, 1983). The temperate population of Europe is a typical B-migration strategist species, having shorter stretches of flight with more stopover sites, thus minimizing energy expenditure (Wlodarczyk et al., 2007). Minias et al. (Minias et al., 2010) suggests a more complex migration pattern, but the late leaving of breeding grounds with an early returning in spring is in line with the B-strategy. This gives times for the juveniles to fully develop and start the migration together with the adults (Wlodarczyk et al., 2007).

*G. gallinago* has an exceptionally long bill relative to its body size. This allows the bird to probe in deeper waters, commonly seen with its head fully submerged, and the lower body also being in water. Rarely takes food found on the surface, relies mainly on the sense of touch when foraging. This habit of foraging in deeper parts of the wetland is associated with different preys. Studies show that most commonly they feed on earthworms (e.g.: *Lumbricus, Allolobaphora, Eiseniella*) and larvae, however they do consume insects in adult their adult forms. *T. glareola*’s diet consists mainly of insects, especially beetles (e.g.: *Curculionidae, Carabidea, Hydrophylidae, Geotrupidae*), however they do feed on larvae and worms in smaller quantities (Cramp & Simmons, 1983). These differences may result in different exposure to certain bacterial taxa, as there are greatly different conditions near the surface and deep in the wet soil.

### 2.2 Sample collection and sequencing

Our sample collection site is located at Andaháza, near Berettyóújfalu, a reconstructed shallow water habitat in the area of the Hortobágy National Park Directorate (HNPD), Hungary. Sampling took place between 27/07/2023 and 25/10/2023 from birds captured with mist nets. After sampling, the birds were released in the shortest possible time to reduce the stress caused by the handling. During this period, we collected a total of 54 samples, of which 33 were from *G. Gallinago* and 21 from *T. glareola*.

The birds were captured using ethical methods accepted and approved by the Ornithological and Migration Research Department of the Hungarian Ornithological and Nature Conservation Society. The bird capture, handling, and sampling protocols were approved under permit numbers PE-KTF/97-13/2017 and PE-KTFO/3533-11/2021, in accordance with Workplace Animal Welfare Committee at the HUN-REN Centre for Ecological Research, the committee of Environmental and Nature Conservation Directorate of the Pest County Government Office (Pest Megyei Kormányhivatal Környezetvédelmi és Természetvédelmi Főosztály) and Ornithological and Migration Research Department of the Hungarian Ornithological and Nature Conservation Association.

We collected cloacal swabs from all captured birds, as this method is more reliable than fecal sample collection for ensuring consistent sampling across individuals. Each swab was placed in 1 ml of Zymo DNA/RNA Shield™ Swab Collection Tube (Zymo Research) and stored at room temperature until DNA extraction. DNA was extracted using the ZymoBIOMICS™ DNA Miniprep Kit (D6010, Zymo Research), following the manufacturer’s protocol optimized for faecal and soil microbiome samples.

To achieve high-resolution taxonomic profiling, we amplified and sequenced the full-length 16S rRNA gene using the Oxford Nanopore 16S Barcoding Kit 1–24 (SQK-16S024). The universal bacterial primers 27F (5’-AGAGTTTGATCMTGGCTCAG-3’) and 1492R (5’-TACGGYTACCTTGTTACGACTT-3’) – included in the sequencing kit - were used, targeting the V1–V9 regions of the 16S gene. PCR amplification was performed using the LongAmp® Hot Start Taq 2X Master Mix (New England Biolabs, M0287). Thermal cycling conditions consisted of an initial denaturation at 95°C for 60 s, followed by 25 cycles of denaturation at 95°C for 20 s, annealing at 55°C for 30 s, and extension at 65°C for 2 min, with a final extension at 65°C for 5 min.

The libraries were sequenced with the MinION Mk1C platform using R9.4 flowcells. We controlled the quality of sequencing experiments by using sterile nuclease-free water as negative and the ZymoBIOMICS Microbial Community DNA Standard D6305 (Zymo, Irvine, California, USA) as a positive control.

### 2.3 Data analysis

We base called the raw sequencing data with Guppy 6.4.6+ae70e8f (Oxford Nanopore Technologies, Oxford, UK) using the super-high accuracy model to obtain high-quality sequencing reads. We then filtered the sequencing reads to remove those with a quality lower than 8 and shorter than 1200 bp or longer than 1800 bp using NanoFilt 2.8.0 (De Coster et al., 2018). We classified the reads with emu 3.4.4 (Curry et al., 2022) using the standard emu database and then normalised the read counts and abundances of taxa by the median 16S gene copy numbers of the species obtained from rrnDB (Stoddard et al., 2015) to reduce the copy number bias of metabarcoding experiments.

We now provide all metadata and descriptive statistics of reads as a part of the Supplementary material in tabular format. Moreover, we provide the output of NanoPlot both for the raw and filtered dataset to evaluate the descriptive statistics graphically. Regarding contamination, the negative controls yielded only a small number of reads and taxa, suggesting that background contamination was minimal in this dataset. We therefore did not observe evidence for substantial contaminant signal that would justify additional decontamination filtering. Importantly, no samples and no taxa were excluded on the basis of contaminant removal. Given the low biomass nature of cloacal swab samples, we treated the positive and negative controls as a quality check; because negative controls contained very few reads and did not indicate a strong contamination signal, we did not apply further contaminant filtering beyond the standard QC and negative-control monitoring.

We excluded reads that could not be assigned to sample, or which we were unable to classify to any bacterial taxa. The remaining sequencing data was used for downstream analysis. The scripts used for the analysis are provided in the Supplementary materials, to further strengthen reproducibility.

**Fig. 1:**
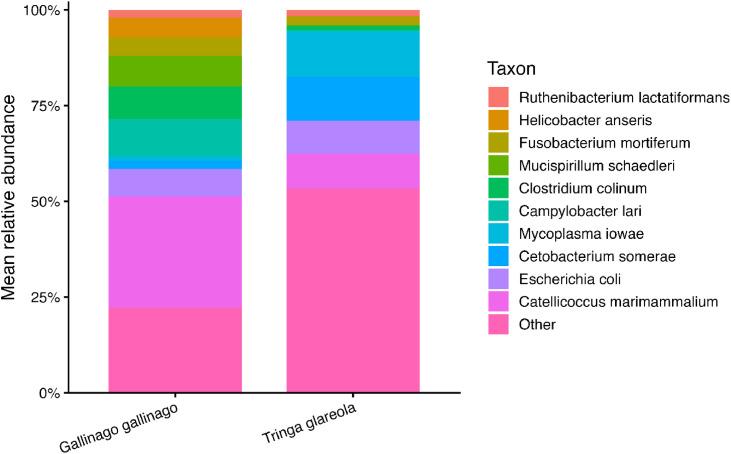
Stacked bar plots show the mean relative abundance (%) of bacterial taxa in the two species. The ten most abundant bacterial taxa are indicated separately, with the rest grouped as “Other”. Taxa are distinguished by fill colour.

All statistical analyses and visualisations were performed in R version 4.4.1 using the packages ‘vegan 2.8’, ‘ggplot2 3.5.2’, ‘patchwork 1.2.0’, ‘rstatix 0.7.2’, ‘dplyr 1.1.4’, ‘tidyr 1.3.1’, and ‘stringr 1.5.1’, ‘stats 4.5.2’, ‘cowplot 1.2.0’, ‘scales 1.4.0’, ‘forcats 1.0.1’, ‘readr 2.2.0’ (Core Team, 2025; Hadley Wickham, 2016; Kassambra A, 2023; Oksanen et al., 2025; Pedersen, 2019; Wickham H, 2025b, 2025a; Wickham H et al., 2026; Wickham H, Francois R, et al., 2025; Wickham H, Pedersen T, et al., 2025; Wickham H, Vaughan D, et al., 2025; Wickham et al., 2025, Wilke, 2025). Microbial community composition data were processed to remove samples with zero total abundance, and abundance values were fourth root transformed before multivariate analyses to reduce the influence of dominant taxa. We used PERMANOVA (*adonis2*, 999 permutations) to test whether gut microbial communities differed significantly between the two host species. We visualized the differences in the microbiome community composition between the two bird species using Principal Coordinates Analysis (PCoA) based on Bray-Curtis dissimilarities calculated from fourth-root transformed species abundance data (Fig. 4). Taxon abundance data were fourth-root transformed to moderate the influence of dominant taxa on Bray–Curtis dissimilarities, allowing differences among less abundant taxa to contribute more strongly. The Bray-Curtis dissimilarity matrix was generated using the *vegdist* function and the ordination was performed using classical multidimensional scaling (*cmdscale*). The same Bray-Curtis matrix was used for PERMANOVA and tests of homogeneity of multivariate dispersions. To test for differences in multivariate dispersion (β-diversity), we applied the *betadisper* function to the Bray-Curtis dissimilarity matrix, followed by ANOVA and Tukey HSD post hoc tests. To explore taxa contributing most to the average Bray-Curtis dissimilarities, we performed a SIMPER analysis using the *simper* function. The p-values for individual microbial species were generated using the permutation implemented in the *simper* function (999 permutations). No correction for multiple testing was applied. To assess within-sample (α) diversity, we calculated Shannon diversity (H′), Simpson diversity (1 – D), inverse Simpson (1/D), and species richness (S) using functions from the ‘vegan’ package. Simpson’s index was computed explicitly as 1 – Σ pᵢ² (where pᵢ is the proportional abundance of each taxon) to ensure that higher values indicate greater evenness. Comparisons of α-diversity between *G. gallinago* and *T. glareola* were conducted using two-sided Wilcoxon rank-sum tests, and effect sizes (rank-biserial correlation r) were obtained with *wilcox_effsize()* from the ‘rstatix’ package. Diversity patterns were visualised using boxplots with jittered points, annotated with exact p-values for each index (Fig. 1). The core microbiome was identified separately for each host species using the prevalence–abundance approach. Taxa were defined as part of the core microbiome if present in ≥ 80 % of individuals and with relative abundance ≥ 0.1 % in those individuals (Astudillo-García et al., 2017; Kassambra A, 2023; Risely, 2020). Mean relative abundances and prevalence values were then visualised, and prevalence being represented by a colour gradient.

## 3 Results

### 3.1 Microbial community composition

We found substantial difference in the composition of the gut microbiome of the two host species. From *G. gallinago* 745 bacterial species were identified in the 33 samples, while 640 species were found in the 21 samples collected from *T. glareola.* 276 of these species were identified in both hosts, thus a total of 1109 species from 18 bacterial phyla was identified. The *T. glareola* samples were dominated by *Proteobacteria* (29.8%) and *Fusobacteria* (29.4%), along with *Firmicutes* (18.0%), *Tenericutes* (14.7%), *Chlorobi* (4.5%) and *Bacteroidetes* (1.4%). In the *G. gallinago* samples, the most abundant phyla were *Firmicutes* (42.0%), *Proteobacteria* (32.1%), *Deferribacteres* (10.6%) and *Fusobacteria* (8.8%), however *Sphirochaetes* (3.1%), *Actinobacteria* (1.6%) and *Tenericutes* (1.5%) also exceeded 1% of all identified bacteria (Fig. 1). We were unable to assign 2.1% of all reads to bacterial taxa.

The PERMANOVA model revealed a significant effect of bird species on community structure (R² = 0.094, F = 5.37, p = 0.001), suggesting that microbial assemblages differ significantly between the two host species. To determine whether this difference may also be affected by heterogeneity in within-group dispersion, we assessed the homogeneity of multivariate dispersion using the betadisper function. Pairwise comparison showed that *T. glareola* had significantly higher within- group variability than *G. gallinago* (mean distance to median = 0.5311 vs. 0.5860, F= 4.56 adjusted p = 0.037). These results suggest that the significant PERMANOVA result should be interpreted with caution, as the observed differences between the two bird species likely reflect both shifts in community composition (centroid location) and differences in dispersion, with *T. glareola* having a more heterogeneous gut microbiota.

As an exploratory approach the SIMPER analysis revealed multiple taxa contributed to the average dissimilarity between *G. gallinago* and *T. glareola*. The major contributor was *Catellicoccus marimammalinum* (mean contribution = 2.76 %, p = 0.003), which was much more abundant in *G. gallinago* (mean relative abundance = 22.76 %) than in *T. glareola* (8.27 %). Other important representatives were *Clostridium colinum* (mean contribution = 2.27%, mean relative abundance = 15.37% vs. 2.69%, p = 0.001), *Campylobacter lari* (mean contribution = 1.88%, mean relative abundance = 12.11% vs. 2.46%, p = 0.042) and *Mucispirillum schaedleri* (mean contribution = 1.76%, mean relative abundance = 11.86% vs. 0.79%, p = 0.041). Other taxa with lower abundances, such as *Cetobacterium somerae, Enterococcus faecium* and *Campylobacter jejuni* also contributed significantly to the dissimilarity between species despite their relatively low mean abundance.

### 3.2 Microbial variability within the host and description of the core microbiome

Permutation tests on Bray-Curtis dissimilarities within species revealed contrasting patterns of microbial community stability between the two bird species. In *G. gallinago*, the mean pairwise dissimilarity between individuals was 0.765, well below the value that would be expected if samples were randomly assigned to species (p < 0.001), suggesting that individuals have a more similar gut microbiome than would be the case by chance. In contrast, *T. glareola* showed a mean within-species dissimilarity of 0.850, which was not significantly different from random expectation (p = 0.774), indicating high interindividual variation in community composition.

**Fig. 2:**
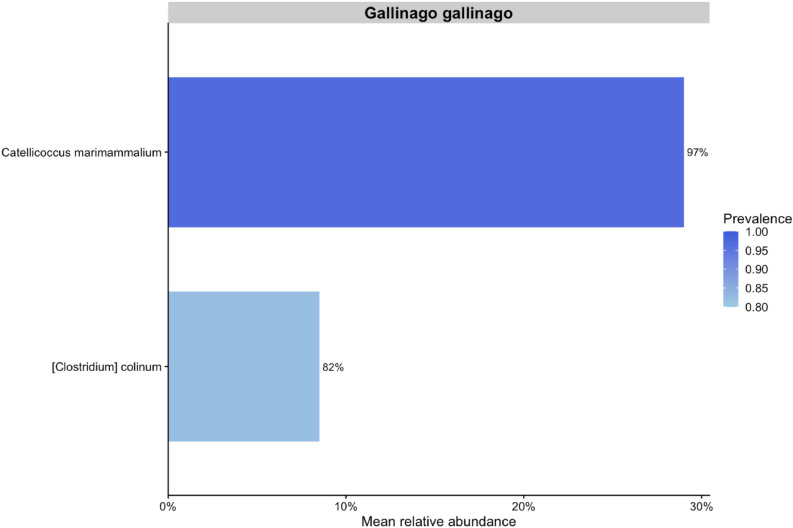
The bars show the mean relative abundance (%) of the main bacterial taxa for the snipe, defined as taxa that occur in at least 80 % of individuals within the species and have a relative abundance of ≥ 0.1 % in these individuals. The bars are ordered according to the mean relative abundance within the host species. The colour of the fill represents the prevalence (proportion of individuals in which the taxon was detected above the threshold), with darker shades indicating higher prevalence. No bacterial taxa met the described criteria in the Wood sandpiper; thus, we excluded it from the figure.

We defined the core microbiome for both bird species as taxa that occur in at least 80 % of individuals within the species and whose relative abundance in these individuals exceeds 0.1 %. These criteria were met only in *G. gallinago*, by two bacterial species: *Catellicoccus marimammalium* (97.0% prevalence; mean relative abundance 29.0%) and *Clostridium colinum* (81.8% prevalence; mean relative abundance 8.5%) (Fig. 2).

### 3.3 Microbial diversity differences between G. gallinago and T. glareola

**Fig. 3:**
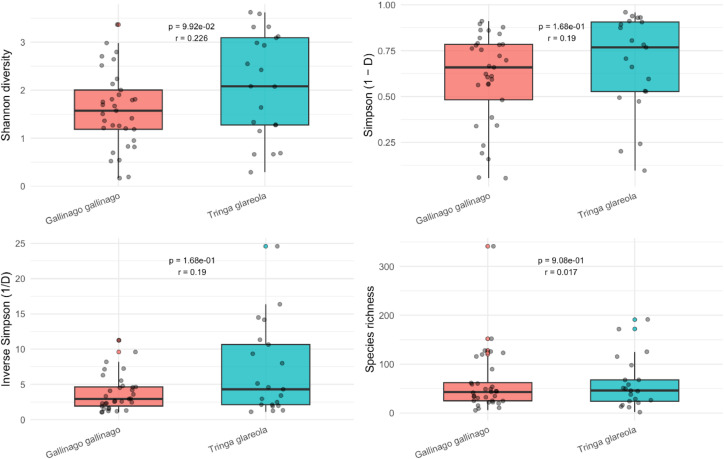
Diversity violin plots with box plots showing the diversity of microbial communities associated with *Gallinago gallinago* (n = 33) and *Tringa glareola* (n=21).

To assess host-associated microbial diversity, we compared α-diversity indices between *G. gallinago* and *T. glareola* (Fig. 3). None of the diversity indices differed significantly between species. Shannon diversity (W = 253, p = 0.099) and Simpson’s diversity (W = 268, p = 0.168) were marginally higher in *T. glareola*, suggesting a trend toward a more even microbial community, but these differences were not statistically significant. Inverse Simpson’s index showed the same tendency (W = 268, p = 0.168), while observed richness was nearly identical between species (W = 340, p = 0.908). Overall, both bird species hosted microbiota of comparable diversity and richness, with only weak evidence of greater evenness in T*. glareola*.

**Fig. 4:**
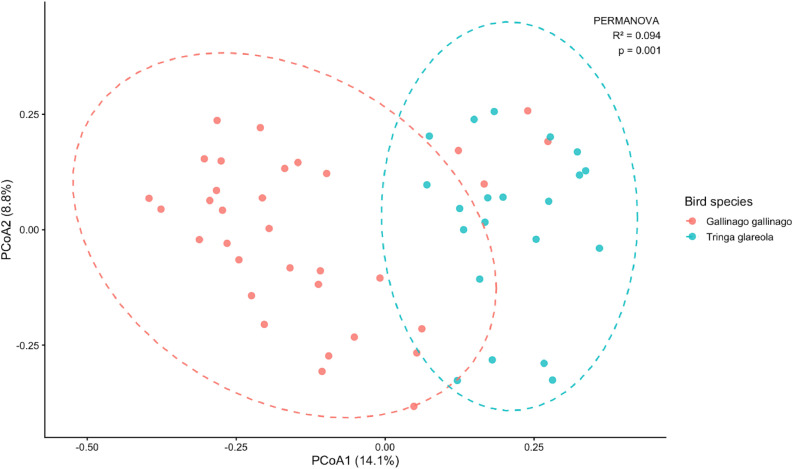
Principal Coordinates Analysis (PCoA) ordination of microbiome communities from the two shorebird species, *Gallinago gallinago* and *Tringa glareola* based on Bray-Curtis dissimilarities of fourth-root transformed abundance data. Each point represents a sample and colours indicate bird species. Ellipses represent the multivariate dispersion of samples within each group. The proportion of variation explained by each axis is shown in parentheses. Community composition differed significantly between bird species (PERMANOVA; see Results).

## 4 Discussion

Studies previously revealed the strong effects of diet and environment on the avian gut microbiome (Bodawatta et al., 2021; Schmiedová et al., 2023). Both hosts being aquatic predators with similar habitat may account for the fact that we did not observe significant difference in microbial richness and alpha- diversities between the hosts. *T. glareola* showed a more heterogenous gut microbiome, with a more even composition, but this was not significant. A potential explanation is the more diverse diet of *T. glareola* is parallelling human data, where a significant relationship had been shown between diversity of diet and the evenness of the gut microbiome (Amamoto et al., 2022). However, these finding should be handled with caution because of the known differences in microbiomes of mammals and birds, while also the shown alpha- diversity differences were not significant in our study. Our general understanding of the avian gut microbiota is insufficient to draw conclusions from small amounts of data. To explain the differences between the two host’s microbiome, there is a need for further research, as there are many variables that this study was unable to address.(Song et al., 2020).

Of the 18 identified phyla one was the Candidate phylum TM6 [*Dependentiae* phyl. nov. (Yeoh et al., 2016)], which is not formally recognised as a phylum yet. It was represented by *Candidatus Babela massiliensis,* an obligate intracellular parasite of *Acanthamoeba castellanii* (Pagnier et al., 2015). Most likely it is not a part of the host’s microbiome, only a transient species from the environment, swallowed with the prey, which does not colonize the gastrointestinal tract of the bird.

A unique gut microbial composition had been found in many different studies of migratory shorebirds, with a high relative abundance of *Fusobacteria.* Multiple studies had described a mean abundance above 10%, at multiple geographic locations, with some reporting a dominance of over 40% during the spring migration (Grond et al., 2019, 2023; Risely et al., 2018; Zhang et al., 2021). On the contrary, Zhao et al. (2022) described a relative abundance of less than 1% across three groups of migratory birds, including waders, raptors and waterfowls. A high abundance of Fusobacteria had been found in different carnivorous groups along with vultures and alligators (Zhang et al., 2021). This dominance had not been described in studies of migrating *Passerines*, despite the clear effect of the migration (Bodawatta et al., 2021; Kreisinger et al., 2015; Trevelline et al., 2023). This may indicate that the migration is not the main driver in the development of the *Fusobacteria* community. Diet and foraging ecology are well-known components in determining birds’ microbiome composition (Bodawatta et al., 2021, 2022). These external factors may give some explanation on our results, as we found a great difference between the two host’s *Fusobacteria* abundances. In the *G. gallinago* samples, only 8.8% of all bacteria belonged to this phylum, in contrast with the 29.4% found in *T. glareola*. The foraging technique is known to present differences between the two species, with *G. gallinago* using its long bill to probe in deeper parts of the sediment, while *T. glareola* relies more on visual prey discovery and probes in shallower parts of the water (Cramp & Simmons, 1983; Durell, 2000; Krupa et al., 2009). The pecking foraging technique suggests a greater proportion of insects in its diet relative to *G. gallinago*. This difference could indicate a higher proportion of chitin in the diet of *T. glareola*, which may account for some of the difference observed. Furthermore, a higher degree of chitin degradation had been described in the shallower parts of the sediment. When the degradation of chitin introduces N-acetylglucosamin (GlcNAc) to the area, a considerable jump in *Fusobacteria* abundance had been described (Wörner & Pester, 2019). This could indicate a more frequent exposure to *Fusobacteria* in the case of *T. glareola*, which would give a possible explanation for the difference observed, however, to strengthen this hypothesis, further testing is needed.

*Catellicoccus marimammalium* is a species of *Firmicutes* commonly found in faecal samples of gulls and waterfowl, with abundances of over 50% across multiple studies (Koskey et al., 2014; Merkeviciene et al., 2017). Although the relative abundances were lower in other groups of birds, *C. marimammalium* can be found in a variety of hosts. Our SIMPER analysis revealed a relative abundance of 36.14% in *G. gallinago* and 8.96% in *T. glareola* samples, which show a similar prevalence with the findings of Ryu et al. (2014), where they found the abundance of the genus *Catellicoccus* to range from 4.8% to 19.1% among three species of migratory shorebirds, during their sampling on stopover sites. In a study conducted on Whooper swans (*Cygnus cygnus*) a substantial difference was found between two sampling sites, which suggests a strong environmental influence in swans (Wang et al., 2021). However, there are signs which makes a strong symbiosis with the host a likely possibility, which may be the case for shorebirds and gulls. *C. marimammalium* is hard to grow on synthetic media, which, together with other findings, may show a better adaptation to living in the gut, rather than in the environment. Reduced metabolic pathways had been discovered, with a highly limited capacity for *de novo* amino acid biosynthesis relative to closely related taxons. The specialized nutrient transport also suggests a symbiotic lifestyle (Weigand et al., 2013). Despite gulls being the most recognised hosts of *C. marimammalium* (Koskey et al., 2014; Merkeviciene et al., 2017; Weigand et al., 2013), notable abundances had been described among different shorebirds, swans and passerines (Ryu et al., 2014; Schmiedová et al., 2023; Trevelline et al., 2023; Wang et al., 2021). In tropical *Passerine* hosts, the abundance of the genus *Catellicoccus* was significantly higher during the rainy season, the difference with dry season being bigger, than between dry season tropical, and temperate species’ samples (Schmiedová et al., 2023). Travelline et al. (2023) revealed a decline of *Catellicoccaceae* during migration in Blackpoll Warblers (*Stretophaga striata*) which indicates a looser relationship with the host, at least in passerines. *C. marimammalium* is more common in species with habitats closely intertwined with wet areas as well as in wet seasons. To elucidate the reason behind the strong relationship with shorebirds, gulls and waterfowl there is a need for researching possible phylosymbiosis, environmental factors, or if it is connected to the lifestyle and body morphology.

A multitude of animal and human pathogens were identified, which could potentially be transported with these avian hosts. It has been found that there is potential for transmission between wild birds and domesticated animals such as poultry or cattle through contamination of feeding stations, drinking water and grazing grounds. Waterbirds also frequent reservoirs in flocks, especially in the winter, thus growing the possibility of contamination of the water reserves (Benskin et al., 2009). For the discussion on the following bacteria the limitations of 16S metabarcoding should be acknowledged. This technique can only identify species; however, it is unable to give information on the strain- level identity, viability, anti-microbial resistance or other important risk factors for human health. Despite these limitations, the presence of these taxa should be mentioned as potentially important factors for human and environmental health. Among the animal pathogens found in the samples there were bacteria infecting both mammalian and bird hosts. Multiple species had been identified as pathogens of poultry, as it is the case for *Mycoplasma iowae*, which causes embryo mortality in the late stages of incubation in turkeys (Al‐Ankari & Bradbury, 1996). *Brachyspira intermedia* can also cause reproduction problems in poultry, as it’s infection causes a lowered egg production in chicken (Feberwee et al., 2008). Multiple pathogens were found which can cause different gastrointestinal problems in birds and mammals; *Helicobacter pullorum*, causing diarrhoea in poultry and in humans, *Clostridium colinum* causing ulcerative enteritis in poultry and *Brachyspira hyodysenteriae*, which causes swine dysentery, a severe enteric disease of pigs (Burrough, 2016; Javed et al., 2017; Kondo et al., 1988). We identified several different human pathogens, many of which were found in high abundances. *Escherichia coli* and *Enterococcus faecalis* are commonly found in the human gastrointestinal tract, however they can be pathogenic, while they are known to be often resistant to a range of antibiotics (Boeder et al., 2024; Erb et al., 2007; Kaper et al., 2004). In multiple samples we identified the opportunistic human pathogenic *Citrobacter freundii*, which had been described to be increasingly resistant to beta-lactams (Jabeen et al., 2023; Pepperell et al., 2002). A possible long-range dispersion of these antimicrobial resistance genes by the hosts could accelerate the growing problem of antibiotic resistance worldwide. Although the species mentioned are known to be resistant to antibiotics in some instances, it is important to note, that our research did not investigate this directly, thus we present this information only as a potential risk. Among the common human pathogens found was *Campylobacter jejuni* and *C. coli* which are common causes of enteric illnesses, with *C. lari* also sporadically causing symptoms in humans (Martinot et al., 2001; Snelling et al., 2005; Tam et al., 2003). A common organism in freshwater habitats, which we identified in 23 out of 54 samples is *Aeromonas veronii*, a possible endosymbiont of the Medicinal leech (*Hirudo medicinalis*) (Graf, 1999). Medicinal leech is most likely part of the diet of both hosts, which further helps with dispersing the bacteria. *Aeromonas veronii* is also pathogenic to humans (Li et al., 2020). We found *Vibrio cholerae*, a common pathogen in wet environments, in low relative abundances in six samples and it was revealed in both hosts. This presence is likely due to the widespread presence of these bacteria in wet environments, thus can be easily ingested by the birds while foraging. The *V. cholerae* communities found in Hungary and in the region are non-O1/non-O139, nontoxigenic strains, thus are not able to cause cholera (Henczkó et al., 2025), however, may be agents in wound or soft tissue infections in humans (Henczkó et al., 2025; Schirmeister et al., 2014) and is thought to be able to survive for longer periods outside of the host body (Cottingham et al., 2003). *V. cholerae* may be an increasing threat to human health, as it had been shown to withstand higher water temperatures and salinity well, thus it may spread better, as anthropogenic climate change advances (Franco et al., 1997; Henczkó et al., 2025; Pascual et al., 2002; Rehm et al., 2023). Understanding the differences between hosts in their capability to disperse certain pathogens is crucial, to minimalize the health risks posed by migratory birds. To assess these risks, a deeper understanding of the microbiomes, the pathogens viability and a detailed description of the bird- human interactions will be needed. Establishing reference communities and understanding, how pathogens interact with different behaviours of birds will be an important step to effectively counter these potential hazards.

Our results supported some of the previous findings on the topic, such as the high variability of birds’ gut microbial communities, the high abundance of *Fusobacteria* in shorebirds, and that they have the potential for pathogen dispersal during their migration (Bodawatta et al., 2022; Hubálek, 2004; Ryu et al., 2014; Zhang et al., 2021). We found that small differences in diet, or foraging technique may lead to substantial differences in their microbiome composition, however there are some overarching trends among shorebirds. Replicating the tendencies observed in species with similar ecological niches in vastly different parts of the world is important in understanding the factors which determine the composition of the microbiome. Deeper analysis of the phylogenetical and ecological patterns will be required in the future to fully understand the role of these factors.

Our research had multiple limiting factors, which should be considered while evaluating the results. Our sampling was conducted in a single geographic location, during a three-month period. This limits the potential of our results to be applied more broadly, as timing and location may be a highly influential factor. We also used cloacal swabs, to represent the gut microbiome, which is known to not be a highly precise representation of the gut microbial communities, however it is a widely accepted method in the field for the characterisation of the microbiome (Videvall et al., 2018). We did not use direct dietary and environmental data for our comparison, which may hide the influence of the immediate environment. Differing and relatively small sample sizes and the different dispersions observed may also limit the strength of our findings.

Despite the limitations, our research brought in novel approaches and viewpoints, which were rarely used before in microbiome studies. We selected two ecologically closely connected species for the comparison, to provide a strong framework for the interpretation of our results. The sampling was also conducted during a single autumn migration period, helping with the reduction of bias introduced by timing, as seasonality is known to affect the microbiome. This way we have the opportunity, to connect microbial characteristics to certain traits or behaviours of the host. The study also revealed potential links between shorebirds and public health, highlighting the strong One Health relevance of microbiome studies. This became possible because of the availability of long read sequencing, which allowed the high taxonomic resolution achieved, allowing the identification of bacteria on a species level.

## Consent for publication

All authors listed on the manuscript have consented to the submission of this manuscript for publication in Animal Microbiome, and each participated in its development as stated in Author Contributions.

## Statement for study in animals

Our study titled: “Microbiota shows major difference in case of two shorebirds”, we conducted field sampling of the birds using cloacal swabs for microbiome analysis.

During this procedure all necessary permits were given by the local authorities, and the sampling was approved by the Ornithological and Migration Research Department of the Hungarian Ornithological and Nature Conservation Society.

All relevant permit numbers are referenced in the manuscript.

## Funding

This work was supported by national scientific research programs (OTKA-FK 138698) and the Hungarian Academy of Sciences Momentum Research Program.

## CRediT authorship contribution statement

**Ákos Őrsi:** Writing – review & editing, Writing – original draft, Supervision, Methodology, Formal analysis, Data curation, Conceptualization. **Levente Laczkó:** Writing – review & editing, Validation, Methodology, Formal analysis, Data curation. **Renáta Bőkényné Tóth:** Writing – review & editing, Methodology. **Csongor Freytag:** Writing – review & editing, Validation, Supervision, Resources, Project administration, Methodology, Conceptualization. **Pál Tóth:** Writing – review & editing, Project administration, Methodology, Conceptualization. **Gábor Simay:** Writing – review & editing, Project administration, Methodology, Conceptualization. **Nándor Szabó:** Writing – review & editing, Methodology, Conceptualization. **Gábor Kardos:** Writing – review & editing, Validation, Supervision, Resources, Project administration, Methodology, Funding acquisition, Conceptualization. **Ádám Lovas-Kiss:** Writing – review & editing, Visualization, Supervision, Project administration, Methodology, Funding acquisition, Formal analysis, Data curation, Conceptualization.

## Declaration of competing interest

The authors declare that they have no known competing financial interests or personal relationships that could have appeared to influence the work reported in this paper.

## Data Availability

All raw data generated for this study were submitted to NCBI under BioProject PRJNA1358826. Sequencing reads are available under accession numbers SRR36073048- SRR36073102. All supplementary material is available at https://doi.org/10.5281/zenodo.20659598
